# Assessing the adaptive role of cannabidiol (CBD) in *Cannabis sativa* defense against cannabis aphids

**DOI:** 10.3389/fpls.2023.1223894

**Published:** 2023-10-17

**Authors:** Jacob MacWilliams, Erika Peirce, William Jacob Pitt, Melissa Schreiner, Tierra Matthews, Linxing Yao, Corey Broeckling, Punya Nachappa

**Affiliations:** ^1^ Department of Agricultural Biology, Colorado State University, Fort Collins, CO, United States; ^2^ Tri-River Area Extension, Colorado State University, Grand Junction, CO, United States; ^3^ Analytical Resources Core-Bioanalysis and Omics, Colorado State University, Fort Collins, CO, United States

**Keywords:** Cannabis sativa, cannabis aphids, cannabinoids, phytohormones, insect performance

## Abstract

*Cannabis sativa* is known for having unique specialized or secondary metabolites, cannabinoids that are derived from an extension of the terpene pathway in the *Cannabis* lineage and includes more than 100 other similar metabolites. Despite the assumption that cannabinoids evolved as novel herbivory defense adaptations, there is limited research addressing the role of cannabinoids in *C. sativa* responses to insect herbivores. Here we investigated the role of cannabidiol (CBD), the predominant cannabinoid in hemp, in plant defense against cannabis aphid (*Phorodon cannabis*), one of the most damaging pests of hemp. We hypothesize that insect feeding may induce changes in cannabinoids as an adaptive strategy for defense. We found that mean fecundity, net reproductive rate (R_0_) and adult longevity of cannabis aphids was reduced on the high cannabinoid cultivar compared to the low- cannabinoid cultivar in whole plant assays. In contrast, supplementation of CBD in artificial feeding assays increased aphid fecundity from day 1 to day 3. Additionally, aphid feeding did not impact cannabinoid levels in leaf tissues with the exception of Δ^9^-tetrahydrocannabinol (THC). This suggests that other cannabinoids and/or metabolites such as terpenes are causing the observed decrease in aphid performance in the whole plant assays. In addition to cannabinoids, *C. sativa* also possesses a range of defense mechanisms via phytohormone signaling pathways that are well described in other plant species. Indeed, cannabis aphid feeding significantly increased levels of the major phytohormones, salicylic acid, jasmonic acid, and abscisic acid, which are known to be involved in plant defense responses against aphid species. These results highlight the interplay between cannabinoid synthesis and phytohormone pathways and necessitate further investigation into this complex interaction.

## Introduction

The evolutionary arms race between plants and their insect attackers has led to the development of highly sophisticated defense system in plants that produce constitutive or induced specialized or secondary metabolites which have toxic, repellent, and/or anti-nutritional effects on the feeding herbivores (Chen, 2008; Howe and Jander, 2008; War et al., 2012; Erb and Reymond, 2019). An estimated 200,000 specialized metabolites have been isolated and identified so far, but it is likely only a small fraction of all the specialized metabolites existing in nature (Kessler and Kalske, 2018). *Cannabis sativa* L. (henceforth, *C. sativa*) is known for having unique specialized metabolites, cannabinoids, that are derived from an extension of the terpene pathway in the *Cannabis* lineage and includes delta-9-tetrahydrocannabinol (Δ^9^-THC) and cannabidiol (CBD), and more than 100 other specialized metabolites (Andre et al., 2016). However, despite the assumption that cannabinoids evolved as novel herbivory defense adaptations, there is limited research addressing the role of cannabinoids in resistance to feeding herbivores, especially the lesser-known cannabinoids. In addition to cannabinoids, *C. sativa* also possesses a range of defense mechanisms (*e.g.* phenolic compounds, protease inhibitors, terpenes) that are conserved across dicots and well described in other model plant species (Kariñho-Betancourt, 2020). Regulation of the specialized metabolites typically occurs through phytohormone signaling pathways are often herbivore-specific and depend on the feeding mode of the herbivore (Walling, 2000). In general, feeding by chewing insects such as caterpillars is mainly regulated by the phytohormone jasmonic acid (JA) (Howe and Jander, 2008). Conversely, piercing-sucking insects such as aphids commonly activate the salicylic acid (SA) pathway instead, which can antagonize the JA pathway (Pieterse et al., 2012; Thaler et al., 2012).

A handful of studies have reported on the negative impacts of cannabinoids on insect herbivores (Rothschild et al., 1977; Rothschild and Fairbairn, 1980; McPartland, 1997). More recent studies have demonstrated the negative impact of CBD on tobacco hornworm (*Manduca sexta*) (Park et al., 2019) and fall armyworm (*Spodoptera frugiperda*) (Abendroth et al., 2023). Feeding by tobacco hornworm decreased CBD production in hemp (Park et al., 2022), which is counter intuitive given the insecticidal property of CBD reported earlier (Park et al., 2019). In contrast, feeding by spider mites (*Tetranychus urticae*) was shown to increase CBD and terpenes (Kostanda and Khatib, 2022). The opposing roles of CBD in pest resistance emphasize the need for a more in-depth study of the role of secondary metabolites in hemp-herbivore interactions. Benelli et al. (2018) showed that essential oil (mainly terpenes) extracted from hemp flowers was toxic to a range of insects including caterpillars, aphids, and mosquitoes. These results highlight the need for further investigation into the role of cannabinoids and other secondary metabolites in multiple pest species to understand the full spectrum of the effects.

Several species of arthropod herbivores have been documented to feed on hemp in the United States (McPartland et al., 2000; Cranshaw et al., 2019). Among them cannabis aphid (*Phorodon cannabis* Passerini), is considered to be one of the most damaging among those that suck plant fluids (Cranshaw et al., 2019). The cannabis aphid is native to Asia and was first reported in North America in 2015. Since its initial detection in Colorado, cannabis aphids have been confirmed in Virginia, Oregon, California, and Ontario, Canada (Cranshaw et al., 2018). Cannabis aphid has often been observed to infest maturing flowers and associated leaflets late in the season and into harvest. In addition to direct damage by ingestion of phloem sap, the aphids vector several plant viruses (McPartland, 1999; McPartland et al., 2000; Pitt et al., 2022). A detailed understanding of plant immunity in a “new” crop such as hemp will provide insights into plant-insect chemical communication and coevolution and facilitate new approaches to crop protection and improvement.

In the current study, we sought to elucidate the adaptive role of CBD in defense against *P. cannabis* in hemp. This was accomplished by the following objectives: 1) assess the life history and behavior of cannabis aphids on high- and low-cannabinoid hemp cultivars, 2) identify the role of CBD, the predominant metabolite in hemp on aphid performance, and 3) identify the impact of cannabis aphid infestation has on plant signaling pathways and cannabinoids in the vegetative stage of *C. sativa*.

## Materials and methods

### Aphid and plant sources

The cannabis aphids used in this study were initially collected from an indoor hemp facility in Loveland, CO. Insects were reared and maintained on Elite (New West Genetics, NWG Fort Collins, CO) cultivar hemp plants in 45.7 × 45.7 × 76.2 cm cage (BioQuip Products Inc., Rancho Dominguez, CA) in a greenhouse at Colorado State University’s Plant Growth Facilities with 430W HPS (High Pressure Sodium) Fixtures (P.L. Light Systems) and 400W bulbs (GE Lucalox lu400 series), photoperiod of 16:8 (L:D) hours (h) and the day: night temperature was 23:18°C.

Hemp cultivars used in this study were high-cannabinoid cultivar, Unicorn (Colorado Hemp Institute, CO), and low-cannabinoid cultivars, Tiborszallasi (European project Multihemp-multihemp.eu) and Elite (New West Genetics, Fort Collins, CO). Unicorn is an Association of Official Seed Certifying Agencies (AOSCA) certified high-CBD cultivar (8-9%, B. Althouse, Colorado Hemp Institute personal communication) and cloned from vegetative cuttings. Tiborszallasi is a European cultivar reported to be a low-CBD (2-3%, Glivar et al., 2020) and is grown from seed. Elite is an AOSCA certified low-CBD cultivar (2-3%, R. Flecther, New West Genetics personal communication) or grain and fiber cultivar and was grown from seed. All cultivars produce less than 0.3% THC. The high-cannabinoid cultivar was cloned from vegetative cuttings and the cuttings were rooted using liquid rooting concentrate (Dip ‘n Grow). The low-cannabinoid cultivars were grown from seed. The plants were grown in insect-proof cages (55 cm x 60 cm x 165 cm) constructed with PVC pipes and covered in fine mesh with an opening to water plants under greenhouse conditions described above. All plants were fertilized with Osmocote (Scott’s Company, Marysville, OH) 15-9-12 N:P:K ratio time-released fertilizer as per label instructions and watered ad libitum.

### Assessing aphid performance on high- and low-cannabinoid cultivars in whole plant assays

To determine the impact of cannabinoids on aphid performance, we analyzed aphid life histories on high- (Unicorn) and low-cannabinoid (Tiborszallasi) cultivars using the insect-proof cages (55 cm x 60 cm x 165 cm) under greenhouse conditions described above. Briefly, two adult aphids were placed in a 1.2 cm clip cage on the abaxial surface of the 3rd or 4th leaf, of a 5-week-old hemp plant with two clip cages were placed on each plant. Clip cages were constructed of 5-mm diameter by 15-mm long clear plastic straw sections with a removable foam top glued to a 10-cm of 20-gauge galvanized steel wire. The adults were allowed to larviposit for 24 h, the adults were removed, and remaining nymphs were left to mature. The number of nymphal instars was checked daily. Once nymphs reached 3rd instar all, but one was removed. The life history of this aphid was monitored daily, and any nymphs produced were removed until the adult died. There was a total of 13 replicates for the high-cannabinoid cultivar (Unicorn) and 21 replicates for the low- cannabinoid cultivars (Tiborszallasi). Aphid observations were conducted in a growth chamber (Conviron, Winnipeg Canada, Model E15) where the photoperiod was 10:14 (L:D) hours (h) and the day: night temperature was 22:18°C.

The life history parameters analyzed are listed in **Table 1**. All formulas used in this study are outlined in **Supplementary Table 1** and based on equations from (Birch, 1948; Chi and Liu, 1985; Chi, 1988; Chi and Su, 2006; Huang and Chi, 2011; Tuan et al., 2014). Data analysis was conducted using the TWOSEX-MSChart (Chi, 2021). The variance and standard errors were calculated using a bootstrap technique. Pairwise comparisons between cultivars were based on a 5% confidence interval using 100,000 bootstrap samples (Efron and Tibshirani, 1993). Figures were generated using R software (R Core Team, 2019, Version 3.6.2) and R package ggplot2 (Wickham, 2009).

**Table 1:** Cannabis aphid reproduction and life table parameters on high and low-cannabinoid hemp cultivars.

Life history parameters	Cultivars	
	High-cannabinoid^a,b^ (Unicorn)	Low-cannabinoid^a,b^ (Tiborszallasi)
Mean fecundity	15.54 ± 3.28 b	39.667 ± 4.83 a
Net reproductive rate (R_0_)	15.54 ± 3.28 b	39.667 ± 4.83 a
Mean generation time	8.85 ± 0.73 b	12.353 ± 0.40 a
Doubling time	2.24	2.33
Developmental Time (days)	6.38 ± 0.59 b	8.33 ± 0.29 a
Adult longevity (days)	5.31 ± 0.94 b	10.19 ± 0.9 a

^a^Values indicate mean ± SE. Standard errors were estimated using 100,000 bootstrap resampling.

^b^Differing letters following means signifies significant differences (*P* < 0.05) based on a paired bootstrap test.

### Assessing aphid performance with cannabinoids in artificial feeding assays

To determine the role of CBD in aphid performance, cannabis aphids were reared on artificial diet supplemented with CBD at 16:8 (L:D) h photoperiod and 22°C under laboratory conditions. The artificial diet was designed for *Myzus persicae* (Mittler and Dadd, 1962). Ten age synchronized 1-day old adult aphids were placed in an artificial feeding chamber consisting of 55 mm Petri dishes (VWR) with parafilm (Bemis) as described in Nachappa et al., 2016. The artificial diet volume was 250 μL including the CBD/DMSO, or DMSO. Stock CBD (Cayman Chemical) solution was prepared by dissolving 10 mg of CBD with 318 μL of DMSO to make 100 mM CBD stock solution. From the 100 mM stock solution, 2.5 μL was used in each feeding chamber for a final 1 mM concentration. A 100 mM CBD was used as stock solution because of previous published reports of using the same concentration (Park et al., 2019). Aphid populations were monitored daily for 4 days. Twelve - fourteen replicates were performed for each treatment, and analysis for each timepoint was done using a generalized linear model (GLM) with a one-way ANOVA followed by Tukey’s HSD test point using R software (version 3.6.2).

### Evaluating aphid preference for high- and low- cannabinoid cultivars in whole plant assays

Cannabis aphid preference for high-cannabinoid (Unicorn) versus low-cannabinoid (Elite) cultivars was measured using whole-plant choice assays similar to that described in Kamphuis et al., 2012. The experiment was conducted at 16:8 (L:D) h photoperiod and 22°C under laboratory conditions. Tiborszallasi was used as a low-cannabinoid cultivar in most experiments, however, our seed stock was depleted, and we could not find a new seed source, so we used another low-cannabinoid cultivar, Elite for the preference study. One 3-week-old plant of both cultivars plant were placed directly across from each other in a 12” cube insect rearing cage (BioQuip) covered in 600 micron light transmitting mesh. A 100 x 15 mm petri dish containing 50 alatae cannabis aphids was placed 10 cm off the ground at the soil level of each pot directly between both hemp plants. The settling of aphids was observed at 1, 2, 3, 6, 24, 48, and 72 hours after release. There were a total of 18 replicates and analysis was performed using Friedman tests for the overall model and Kruskal–Wallis tests for each individual time point using R software (version 3.6.0).

### Analysis of cannabinoids using ultra-performance liquid chromatography tandem mass spectrometry

For cannabinoid analysis, we analyzed 14 cannabinoids in the high- (Unicorn) and low- (Tiborszallasi) cannabinoid hemp cultivars that were either infested with cannabis aphids or left uninfested (control plants). Briefly, 8-week-old plants were infested with 10 mixed life stages of cannabis aphids by placing the aphids on the adaxial surface of nodes 9 – 13. The infested and uninfested plants were housed in separate cages (55 cm x 60 cm x 165 cm). Twenty days post-aphid infestation, three leaves, taken from the two uppermost fully expanded nodes, were harvested from each treatment, and aphids were removed using paintbrushes. The leaves were placed in 50 mL conical tubes and stored at -20 C. There were four replicates collected for each cultivar. Samples were transported to a -80°C freezer for 2 hours immediately before lyophilization. Samples were lyophilized for 49 h. After lyophilization, samples were stored in a -20°C freezer. Lyophilized samples were then homogenized for 5 min using a bead beater (Next Advance, Troy, NY, USA). After homogenization, about 40 mg tissue for each sample was weighed into 2-mL Eppendorf tubes, with 1 mL of cold 80% methanol in water. Samples were then vortexed vigorously for 30 min at 4°C, followed by 15 min sonication in an ice bath and another 30-min vigorous vortexing at 4°C. After mixing, samples were centrifuged at 15,000 g and 4°C for 10 min. Supernatants were recovered and diluted 10 times in cold 100% methanol. Then 50 μL of the diluted sample was mixed with 50 μL of internal standard (IS) and stored at -80°C until analysis. An aliquot (10 μL) was taken from each study sample to be pooled to generate a quality control (QC) sample. The IS, THC-d3 was purchased from Cerilliant (TX, USA), and then diluted in 100% methanol to obtain 0.1 μg/mL for spiking.

UPLC-MS/MS analysis was performed on a Waters ACQUITY UPLC coupled to a Waters Xevo TQ-S triple quadrupole mass spectrometer. Chromatographic separations were carried out on an ACQUITY UPLC HSS T3 column (1 x 100 mm, 1.8 μm, Waters, MA, USA) described in **Supplementary Table 2**. The capillary voltage of MS detector was set to 0.7 kV in positive mode. Inter-channel delay was set to 3 msec. Source temperature was 150°C and the desolvation temperature was 450°C. Desolvation gas flow was 1000 L/h, cone gas flow (nitrogen) was 150 L/h, and collision gas flow (argon) was 0.15 mL/min. Nebulizer pressure (nitrogen) was set to 7 Bar. Autodwell feature was set for the collection of 12 points-across-peak. The cone voltage and collision energy (CE) of each MRM was optimized (**Supplementary Table 3**). Several high abundance compounds (CBDVA, CBGA, CBDA) in the current sample set were analyzed using a “de-optimized” cone and CE voltage. Raw data files were imported into the Skyline opensource software package (MacLean et al., 2010) for processing. Each target analyte was visually inspected for retention time and peak area integration. Peak areas were extracted for target compounds detected in biological samples and normalized to the peak area of the appropriate internal standard or surrogate in each sample. Absolute quantitation of dry weight (µg/g) was calculated using the linear regression equation generated for each compound from the calibration curve (**Supplementary Figure 1**). Cannabinoids were analyzed using R software (version 3.6.0). Outlier tests using a Bonferroni adjustment were run for all cannabinoid levels. Once outliers were determined, instead of removing outliers by metabolite, we decided to remove the entire sample for further analysis. The difference in cannabinoid levels between infested and control plants were analyzed for statistical significance using a *t*-test.

### Analysis of phytohormones using UPLC-MS/MS

The leaf tissue samples for phytohormone analysis were obtained from the high- (Unicorn) and low- (Tiborszallasi) cannabinoid hemp cultivars that were either infested with cannabis aphids or left uninfested (control plants) used for the cannabinoid analysis described above. The phytohormone analysis was conducted following the reference (Sheflin et al., 2019) with modifications described below. Frozen samples were lyophilized, and the dried samples were added with stainless steel balls and homogenized in a Bullet Blender for 2 min. The homogenate (20-30 mg) was weighed into 2-mL glass extraction vials and added with 1 mL of cold 80% methanol in water and 20 μL of internal standard mix containing 200 ng/mL of SA-D4 (Sigma-Aldrich, St. Louis, MO), 200 ng/mL of JA-D5 (Sigma-Aldrich, St. Louis, MO), and 500 ng/mL of ABA-D6 in 50% methanol (Santa Cruz Biotechnology, Santa Cruz, CA)]. The mixture was vigorously mixed for 30 min, followed by 15 min of sonication and another 30 min of mixing. Then the mixture was centrifuged at 3,000 g and 4°C for 15 min. Supernatant (850 μL) was recovered. To the remaining pellets, 1 mL of acetonitrile was added, and the extraction was repeated as described above. After centrifugation, supernatant (850 μL) was recovered and combined with the first aliquot of supernatant, which was then dried under nitrogen, and then resuspended in 100 μL of 50% methanol in water. An aliquot (10 µL) was taken from each study sample to generate pooled quality control (QC) samples. Sample extracts and QCs were stored at -20°C until analysis.

UPLC-MS/MS analysis was performed on a Waters ACQUITY Classic UPLC coupled to a Waters Xevo TQ-S triple quadrupole mass spectrometer. Chromatographic separations were carried out on a Waters UPLC T3 column (2 x 50 mm, 1.7 μM) as described in **Supplementary Table 4**. The mass detector was operated in ESI- mode. The capillary voltage was set to 0.7 kV. Inter-channel delay was set to 3 msec. The source temperature was 150°C, and the desolvation gas (nitrogen) temperature was 450°C. Desolvation gas flow was 1000 L/h, cone gas flow was 150 L/h, and collision gas (argon) flow was 0.15 mL/min. Nebulizer pressure (nitrogen) was set to 7 Bar. The MS acquisition functions were scheduled by retention time. Autodwell feature was set for each function, and dwell time was calculated by Masslynx software (Waters) to achieve 12 points-across-peak as the minimum data points per peak. The retention time, MRM transitions, cone, and collision energy of each compound were described in **Supplementary Table 5**.

Raw data files were imported into the Skyline open-source software package (MacLean et al., 2010). Each target analyte was visually inspected for retention time and peak area integration. Peak areas were extracted for target compounds detected in biological samples and normalized to the peak area of the appropriate internal standard or surrogate in each sample. Absolute quantitation (ng/g on dry weight basis) was calculated using the linear regression equation generated for each compound from the calibration curve. (**Supplementary Figure 2**). The reference standard at various concentrations was mixed with the internal standard in 50% methanol for the calibration curve. Normalized peak areas are plotted against expected concentrations. Phytohormones were analyzed using R software (version 3.6.0). Outlier tests using a Bonferroni adjustment were run for all phytohormone levels. Once outliers were determined, instead of removing outliers by metabolite, we decided to remove the entire sample for further analysis. The difference in cannabinoid levels between infested and control plants were analyzed for statistical significance using a *t*-test.

### Analysis of phytohormone- and cannabinoid-marker genes using RT-qPCR analysis

Ten adult cannabis aphids were caged onto the adaxial side of the most expanded leaf of 3-week-old high-cannabinoid (Unicorn) plant using clip-cages (Bioquip). The cage was positioned to allow access to both the adaxial and abaxial sides for the aphids. After 48 hours, aphids were removed with a fine paintbrush and leaves were collected and flash frozen in liquid nitrogen for RNA extraction. Uninfested control leaves were treated in the same manner. Total RNA isolation was performed following the CTAB-C/I+RNeasy protocol described in Guerriero et al., 2016. Briefly, RNA was isolated from 100 mg of tissue from three-week old hemp plants by grinding the tissue in a mortar and pestle with liquid nitrogen. Next, 2.5% (% w/v) polyvinylpyrrolidone (PVP-40), 2 M NaCl, 100 mM Tris-HCl pH 8, 25 mM EDTA, 0.2% BME were added, and samples were incubated for 10 min at 60°C, vortexed, and centrifuged. The aqueous phase was extracted and precipitated with 2/3 volume of isopropanol for 1 hour at -20°C. Samples were loaded on RNeasy columns (Qiagen) according to the manufacturer’s instructions with the on-column DNase treatment. The elimination of genomic DNA was validated through PCR. RNA samples were quantified using a nanodrop, and 2 μg of cDNA was synthesized from the RNA samples using Verso cDNA synthesis kit (Thermo Fisher) according to the manufacturer’s instructions. We targeted genes in the *CBD synthesis (Cannabidiolic acid synthase, CBDAS)* and phytohormone signaling pathways using primer sets listed in **Supplementary Table 6**. The four genes were chosen because they are known marker genes for each phytohormone pathway and primer sets have been previously published for them in *C. sativa* (Mangeot-Peter et al., 2016; Gao et al., 2018; Balthazar et al., 2020; Fulvio et al., 2021). RT-qPCR was performed in a QuantStudio 3 (Thermo Fisher) with iQ SYBR Green Supermix (Bio-Rad) in a 20 ul reaction using gene specific primers with 3.5 min at 95°C, 40 cycles of 15 s at 95°C, and 60 s at 58°C. Target genes were normalized to *CsClathrin*, and relative expression levels were calculated with 2-ΔΔCt (Livak and Schmittgen, 2001). Two technical replicates and four biological replicates were performed for each sample.

## Results

### Cannabis aphid performance is decreased on the high-cannabinoid cultivar in whole-plant assays

To evaluate the impact of cannabinoids on cannabis aphids, life history assays were performed on high-cannabinoid cultivar (Unicorn) and low-cannabinoid cultivar (Tiborszallasi) under greenhouse conditions. The mean fecundity and net reproductive rate (R_0_) were found to be the same, and they were significantly higher in the low-cannabinoid cultivar, Tiborszallasi, compared to the high-cannabinoid cultivar, Unicorn (**Table 1**, *P* = 0.0001). In contrast, cannabis aphid development time was shorter on the high-cannabinoid cultivar compared to low-cannabinoid cultivar (**Table 1**, *P* = 0.003). However, once the nymph reached adulthood, aphids survived longer on the low-cannabinoid cultivar, compared to the high-cannabinoid cultivar (**Table 1**, *P* < 0.0001).

### Cannabis aphids’ fecundity increased on high-CBD diet in artificial feeding assays

**Figure 1:**
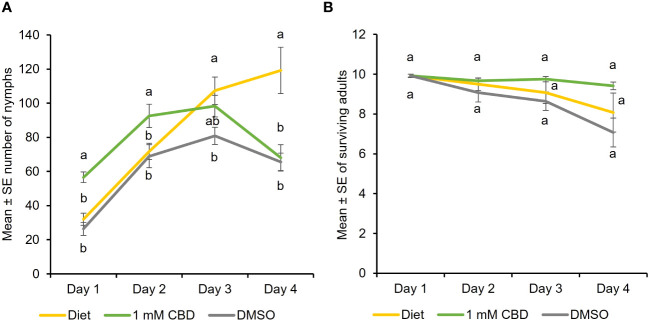
CBD has a positive effect on cannabis aphid fecundity in artificial feeding assays. Cannabis aphids were maintained on artificial diet supplemented with either DMSO or DMSO + 1 mM CBD. A cohort of ten 8-day old adult aphids were transferred to artificial diet and allowed to feed for 4 days and the number of **(A)** nymphs and **(B)** surviving adults remaining each day was monitored (n = 14, n =12 for 1 mM CBD). Analysis with GLM followed by one-way ANOVA with Tukey-HSD *post-hoc* tests were performed for each day. Different lowercase letters indicate significant differences.

To determine the effect of CBD, the predominant cannabinoid in hemp, feeding assays were performed where cannabis aphid was reared on artificial diets supplemented with CBD. Aphid fecundity increased when reared on an artificial diet supplemented with DMSO + 1 mM CBD, relative to diet alone (baseline) and DMSO alone from day 1 until day 3 [Two-way ANOVA: Time (*F *= 21.33, df = 3, *P*<0.0001) and treatment (*F* = 7.52, df = 2, *P* = 0.001); **Figure 1A**). In contrast, the addition of CBD had no significant effect on adult survival at any timepoint (**Figure 1B**). DMSO treatment alone had lowest aphid nymphs and aphid survival indicating DMSO toxicity.

### Cannabis aphids have a delayed preference for low- cannabinoid cultivar compared to high-cannabinoid cultivar

**Figure 2:**
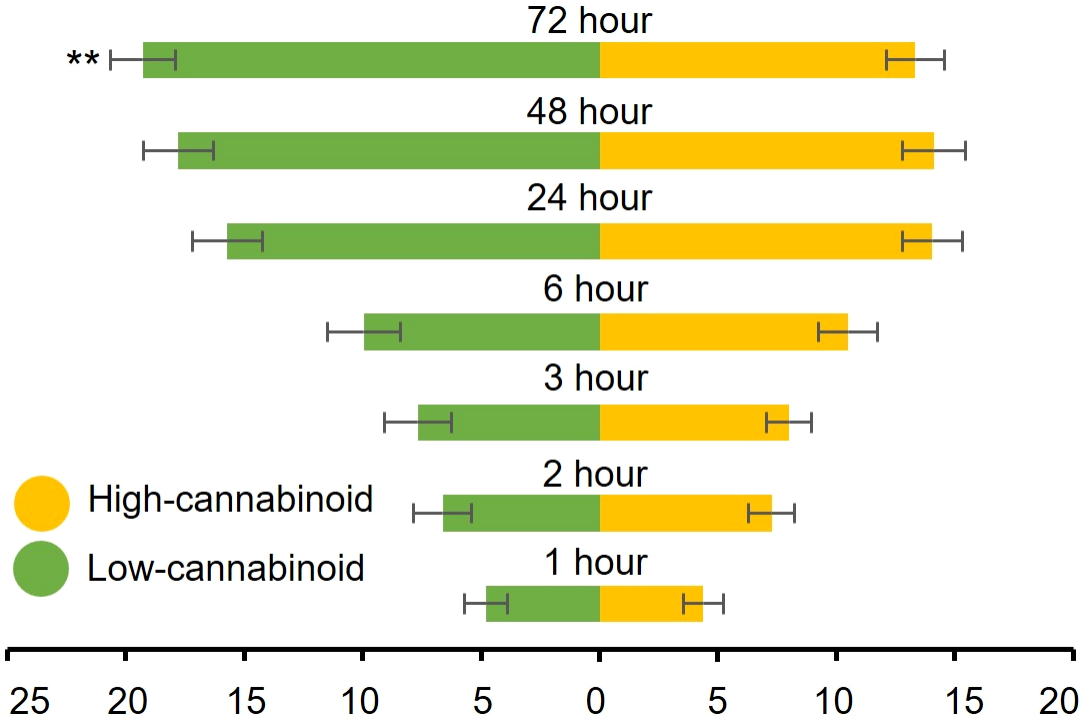
Cannabis aphids have delayed preference for low-cannabinoid cultivar. Fifty alatae cannabis aphids were placed in a Petri dish and introduced to a 12” cube cage to choose between three-week old high-cannabinoid (Unicorn) or low-cannabinoid (Elite) on either side of the cage. The number of alatae on either plant was documented at the indicated timepoints. Values are the mean ± SE of n=18. Analysis with both Friedman (full model) and Kruskal-Wallis (individual time points) tests were performed. Asterisks indicate significance **P < 0.01.

To determine whether cannabinoids have an effect on cannabis aphid preference, fifty alataes were introduced into a cage equal distance between a high-cannabinoid cultivar, Unicorn, and a low-cannabinoid cultivar, Elite, and settling preference was monitored over 72 h. Most of the aphids did not show a preference for either cultivar from 1h through 48h post-release (*H* = 0.10, df = 1, *P* = 0.75; *H* = 0.41, df = 1, *P *= 0.59; *H* = 0.33, df = 1, *P* = 0.57; *H* = 0.44, df = 1, *P* = 0.51; *H* = 2.94, df = 1, *P* = 0.09) (**Figure 2**). However, at 72 h post-release, a significant preference for the low-cannabinoid cultivar emerged (**Figure 2**, *H = *8.436, df = 1, *P* = 0.004).

### Cannabis aphid feeding had minimal impact on cannabinoid production

To determine the impact of cannabis aphid feeding on cannabinoid production, we analyzed cannabinoid production in leaf tissues between cannabis aphid-infested and control (uninfested) or healthy plants using UPLC-MS/MS analysis. A total of 14 cannabinoids were analyzed, out of which nine were detected and quantified, as described in **Table 2**. There was no impact of aphid infestation on cannabinoid concentrations in the high-cannabinoid cultivar (Unicorn) with the exception of increased THC, whereas there was no change in cannabinoid levels in the low-cannabinoid cultivar (Tiborszallasi) (**Table 2**).

**Table 2:** Cannabis aphid impacts on cannabinoid levels in high-cannabinoid cultivar (Unicorn) and low-cannabinoid cultivar (Tiborszallasi).

Cultivar	Cannabinoid	Treatment	Mean ± SE (µg/g dry weight)	t-test_(df)_	*P*-value^a^
High-cannabinoid cultivar (Unicorn)	CBDVA	Control	520.2 ± 45.64	0.28_(8)_	0.39
		Infested	498.3 ± 64.14		
	CBD	Control	10.5 ± 1.27	1.63_(8)_	0.07
		Infested	16.7 ± 3.55		
	CBG	Control	34.1 ± 4.34	0.21 _(8)_	0.42
		Infested	32.7 ± 5.38		
	CBDA	Control	8644 ± 505.57	0.26_(8)_	0.40
		Infested	8425 ± 664.15		
	CBGA	Control	1160 ± 408.37	1.03_(8)_	0.16
		Infested	720.2 ± 120.82		
	THCVA	Control	19.74 ± 1.81	0.08 _(8)_	0.4
		Infested	19.47 ± 2.74		
	Δ^9^-THC	Control	1.426 ± 0.14	1.84_(8)_	**0.05**
		Infested	2.373 ± 0.50		
	Δ^9^-THCA	Control	476.9 ± 41.27	0.18_(8)_	0.43
		Infested	490.7 ± 64.88		
	CBLA : CBCA	Control	1857 ± 146.98	0.38_(8)_	0.35
		Infested	1952 ± 201.06		
Low-cannabinoid cultivar (Tiborszallasi)	CBDVA	Control	95.11 ± 7.65	0.43_(6)_	0.34
		Infested	77.03 ± 41.41		
	CBD	Control	24.35 ± 5.77	0.15_(6)_	0.44
		Infested	23.15 ± 5.37		
	CBG	Control	13.87 ± 1.59	0.26_(6)_	0.40
		Infested	12.77 ± 3.90		
	CBDA	Control	10005 ± 693.20	1.26_(6)_	0.12
		Infested	8162 ± 1286.47		
	CBGA	Control	289.3 ± 157.53	1.60_(6)_	0.07
		Infested	33.17± 26.36		
	THCVA	Control	4.67 ± 0.35	0.60_(6)_	0.28
		Infested	3.84 ± 1.34		
	Δ^9^-THC	Control	3.11 ± 0.75	0.28 _(6)_	0.39
		Infested	3.40 ± 0.70		
	Δ^9^-THCA	Control	582.4 ± 52.63	0.88 _(6)_	0.20
		Infested	485.6 ± 96.94		
	CBLA : CBCA	Control	2908 ± 775.46	1.52_(6)_	0.08
		Infested	1606 ± 365.73		

^a^Bold indicates statistically significant differences.

### Cannabis aphid feeding impacts phytohormone signaling

**Figure 3:**
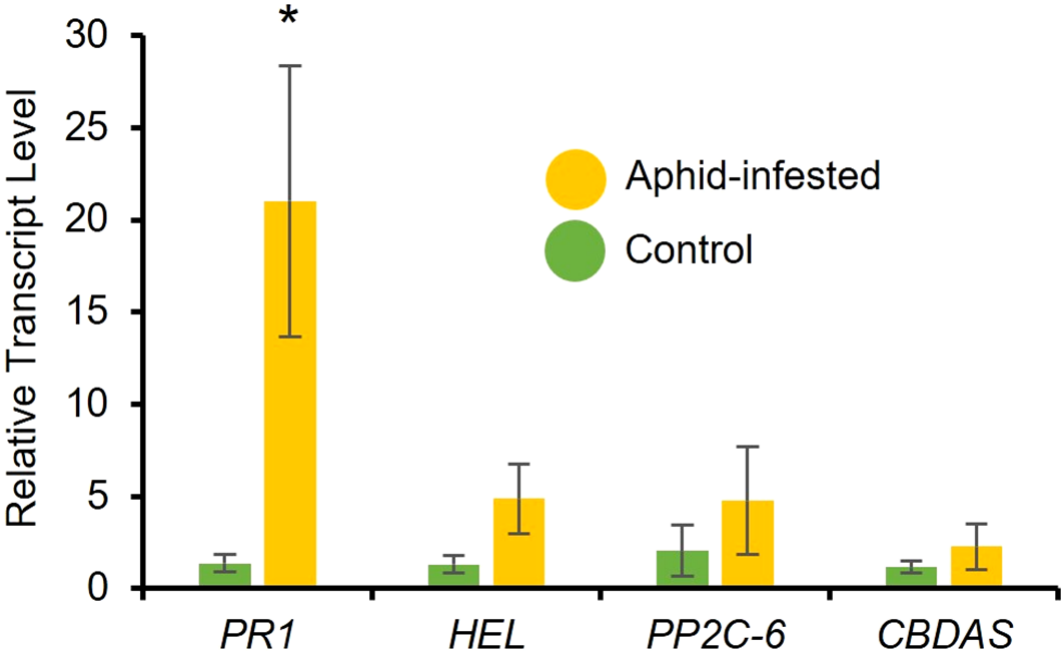
Cannabis aphid infestation induces plant defense responses. Ten adult cannabis aphids were clip-caged on a 3-week-old cannabis single leaf. After 48 hours aphids were removed and leaves were collected and relative expression of phytohormone marker genes for salicylic acid (*PR1*), jasmonic acid (*HEL*), abscisic acid (*PP2C-6*), and CBD (*CBDAS*) were measured with RT-qPCR. Error bars represent SE of the mean of four biological replicates with two technical replicates each. Statistical analysis was performed using a *t*-test. Asterisks indicate significance, *P < 0.05 (t-test).

To determine aphid-induced changes in phytohormone levels, RT-qPCR analysis was performed to determine the expression of marker genes. After a 48-hour infestation period of a 3-week-old high-cannabinoid cultivar (Unicorn), the expression of the SA marker gene, *PR1* was significantly increased in the aphid infested leaf tissues compared to the uninfested controls (**Figure 3**; t = 2.66, *P* = 0.03). While not statistically significant, the JA marker gene *HEL* was also expressed higher at 48 hours (**Figure 3**; t = 1.83, *P* = 0.12) in the aphid infested samples compared to the uninfested controls. There was no effect of aphid infestation on another stress-related phytohormone, abscisic acid (ABA) marker gene *PP2C-6* (**Figure 3**; t = 0.91, *P* = 0.40) and *Cannabidiolic acid synthase* (*CBDAS*) expression (**Figure 3**; t = 0.89, *P* = 0.41) at 48-hour post-infestation.

**Figure 4:**
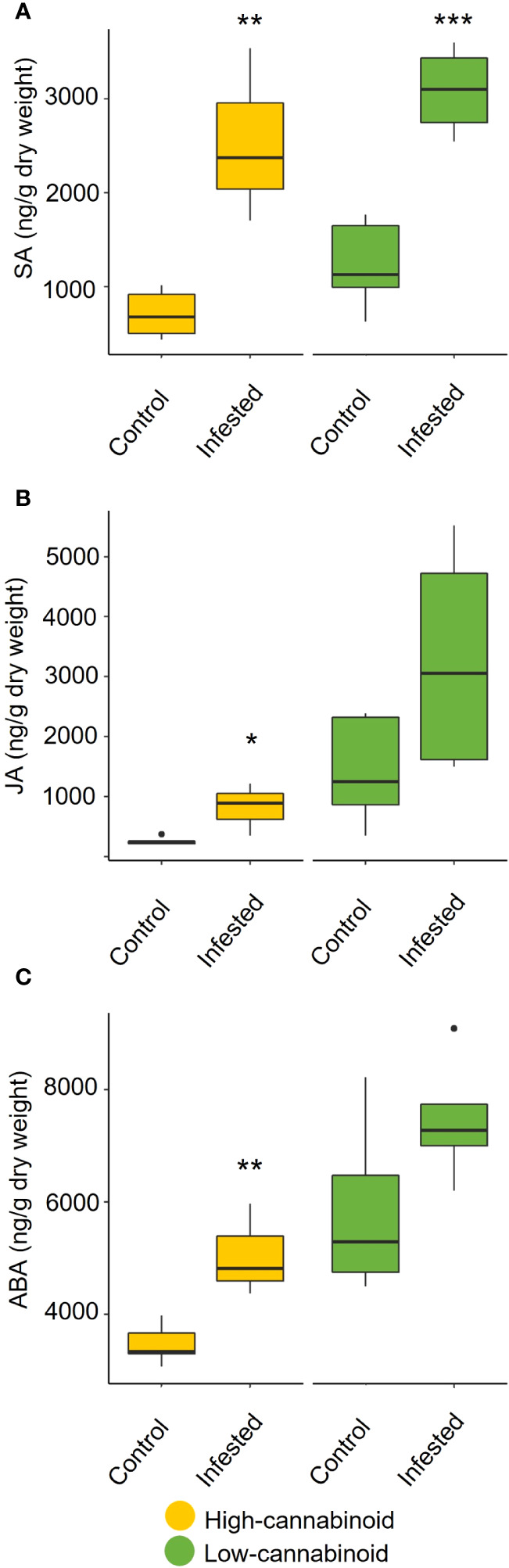
Cannabis aphid feeding impacts phytohormone levels. Eight-week-old hemp plants were infested with 10 mixed life stages of aphids and cannabinoid levels were analyzed 20 days post-infestation. The cannabinoids include **(A)** salicylic acid (SA), **(B)** jasmonic acid (JA), and **(C)** abscisic acid (ABA) production in infested and uninfested leaves for each cultivar. Statistical analysis was performed using a *t*-test. Asterisks indicate significance *P < 0.05, **P < 0.01, ***P < 0.001.

To assess the impact of long-term aphid feeding (20 days) on phytohormone production, the levels of SA and JA, as well as ABA, were monitored on 8-week-old plants using UPLC-MS/MS analyis (**Figure 4**). In the high-cannabinoid cultivar (Unicorn), the aphid infested plants had significantly higher levels of SA compared to the uninfested controls (**Figure 4A**; t = 4.36, *P* = 0.005). This result is consistent with the impact of early aphid infestation as determined by RT-qPCR analysis. The high-cannabinoid cultivar also had higher JA (t = 2.96, *P* = 0.025) and ABA (t = 3.87, *P* = 0.008) levels relative to the uninfested controls (**Figures 4B, C**). In the low-cannabinoid cultivar (Tiborszallasi), aphid infestation only SA levels were significantly impacted by aphid infestation relative to uninfested controls (**Figure 4A**; t = 5.76, *P* = 0.0007).

## Discussion

*Cannabis sativa* possesses a rich diversity of specialized metabolites, including cannabinoids and terpenes, which are known to be involved in the plant’s defense against arthropod pests (McPartland, 1997). Several studies revealed that aqueous, essential oil extracts and solvent extracts of *C. sativa* can also repel insects [Reviewed in (McPartland and Sheikh, 2018)]. However, to date, only a handful of studies have examined the role of specific cannabinoids in defense against insects (Rothschild et al., 1977; Rothschild and Fairbairn, 1980; Park et al., 2019; Abendroth et al., 2023). Hence, there is a need for further investigation into the role of specific cannabinoids and other specialized metabolites in defense against multiple arthropod species to determine the adaptive value of cannabinoids. Here, we demonstrate that cannabinoids reduced the fecundity of cannabis aphid, an invasive piercing-sucking pest of hemp on whole plants; however, supplementation of CBD in artificial feeding assays led to an increase in aphid fecundity. While aphid feeding only had an impact on THC in leaf tissues, aphid feeding significantly increased levels of SA, JA, and ABA in high-cannabinoid cultivar. To our knowledge, this is the first study to investigate the role of cannabinoids, specifically CBD, in *C. sativa* defense against aphids.

To investigate the impact of cannabinoids on aphid performance, a high-cannabinoid cultivar (Unicorn) and a low-cannabinoid cultivar (Tiborszallasi) were used in life history studies. Cannabis aphid life history traits were reduced on the high-cannabinoid cultivar compared to the low-cannabinoid cultivar. This result is in agreement with previous reports of the negative impacts of both CBD and THC on insect performance. For example, the garden tiger moth (*Arctia caja*) had reduced growth and survival on high-cannabinoid cultivars, and the application of CBD and THC was a deterrent of large white (*Pieris brassicae*) oviposition (Rothschild et al., 1977; Rothschild and Fairbairn, 1980). The negative effects have also been observed recently in tobacco hornworm and fall armyworm, where the addition of CBD to an artificial diet led to decreased size and weight in a concentration dependent manner (Park et al., 2019; Abendroth et al., 2023). Though the presence of cannabinoids had a detrimental effect on cannabis aphid performance, development time showed the opposite trend. Cannabis aphids had a shorter development time on the high-cannabinoid cultivar compared to the low-cannabinoid cultivar. Cannabis aphids showed a delayed preference towards low-cannabinoid over high-cannabinoid cultivar leaf tissues in choice assays at 72-hour post-release. Similarly, the tobacco hornworm was also observed to prefer a low-cannabinoid cultivar to a high-cannabinoid cultivar (Park et al., 2019). Interestingly, when cannabis aphids were fed an artificial diet supplemented with DMSO + 1 mM CBD, aphid populations increased relative to diet alone (baseline) and DMSO alone from day 1 until day 3. This suggests that CBD supplementation in artificial feeding assays does not have a negative impact on cannabis aphids that has been observed with other hemp pests like tobacco hornworm and fall armyworm (Park et al., 2019; Abendroth et al., 2023).

Overall, there was minimal effect of aphid infestation on cannabinoid levels. Cannabinoids are synthesized in the secretory cells of the glandular trichomes found most abundantly in female flowers (Potter, 2009) and in low levels in the leaves (Park et al., 2022), seeds (Ross et al., 2000), roots (Stout et al., 2012), and pollen (Ross et al., 2005). Hemp leaves were sampled for RT-qPCR and LC-MS analysis instead of flowers which harbor higher levels of cannabinoids. This may in part explain why no significant difference was observed in cannabinoid levels between aphid and uninfested controls. But we detected increased THC levels in response to aphid infestation in the high-cannabinoid cultivar. Other studies have found that arthropod feeding can alter cannabinoid levels. For example, spider mites’ infestation led to increased CBD and THC levels (Kostanda and Khatib, 2022), whereas tobacco hornworm infestation led to a decrease in CBD and its precursor CBGA (Park et al., 2022). These results suggest that *C. sativa* defense responses are not conserved against herbivores and likely depends on the type (feeding guild) and level (short term vs long term) of stress and interaction with other environmental factors, including abiotic factors. A drawback of our experimental approach is our inability to assign the role of a specific cannabinoid to either insect physiology or plant responses because of the inherent differences in genetic backgrounds of the high- and low- cannabinoid cultivars. Future studies should compare several high- and low-CBD producing cultivars of known pedigrees or utilize knockout near-isogenic lines or exogenously treat low-producing lines with CBD. We are in the process of creating a population of 250 Recombinant Inbred Lines (RILs) from a bi-parental cross of 2 hemp genotypes segregating in parts of cannabinoid, terpene and other putative defense compounds which should help us to begin to address this question.

In contrast to the neutral effect of aphids on cannabinoid levels, there was a strong and positive impact of cannabis aphid infestation on the phytohormone levels. Similar to other aphid-plant interactions, cannabis aphid infestation in hemp leads to the induction of the *PR1*, SA pathway marker gene (Nalam et al., 2019). In addition, cannabis aphid infestation also led to significant increases of SA, JA, and ABA levels in the high-cannabinoid cultivar. Previous research demonstrated that these three phytohormones are known to antagonize each other (Pieterse et al., 2012; Thaler et al., 2012); however, more recent studies have identified increases in all three phytohormones in response to multiple aphid species (Florencio-Ortiz et al., 2020; Koch et al., 2020; Xie et al., 2020). These results suggest the interplay of multiple phytohormones in hemp response to aphid interaction and the need for future transcriptomics studies to better understand the interaction.

There is a growing body of evidence that demonstrates that cannabinoids are differentially affected by phytohormone signaling pathways (Mansouri et al., 2009; Mansouri and Asrar, 2012; Jalai et al., 2019; Mirzamohammad et al., 2021; Apicella et al., 2022). For example, the exogenous application of SA in one study led to an increase in CBD and THC concentrations but in another led to only an increase in THC production and a decrease in CBD production (Jalai et al., 2019; Mirzamohammad et al., 2021). Application of methyl jasmonate (MeJA) to *Cannabis* plants also led to increased cannabinoid production, specifically THC (Apicella et al., 2022). Application of ABA to *C. sativa* plants had differing effects depending on the plant stage when it was applied. In vegetative plants, it reduced both CBD and THC levels, while in mature plants, application of ABA increased THC levels (Mansouri et al., 2009; Mansouri and Asrar, 2012). The contradicting effects between studies necessitate further investigation of the complex interactions between phytohormone and cannabinoid synthesis pathways.

The other predominant metabolite in *C. sativa* is terpenes which could be contributing to the reduced aphid performance on the high-cannabinoid cultivar. Indeed, there are over 100 known terpenes in *C. sativa*, and many are known to act as natural insecticides (Rothschild et al., 2005; Russo, 2011; Benelli et al., 2018). Further studies should investigate the relative contribution of cannabinoids and terpenes in herbivore defense in *C. sativa*. Another aspect that needs to be investigated is the role of trichomes in combating herbivores. *Cannabis sativa* is covered by dense trichomes that may act as a physical barrier to movement and/or secrete specialized metabolites that have anti-herbivory effects. Understanding the impact of *C. sativa* physical and chemical defenses can aid in the development of effective and sustainable pest management program in this new crop, hemp.

## Data availability statement

The original contributions presented in the study are included in the article/**Supplementary Material**. Further inquiries can be directed to the corresponding author.

## Author contributions

JM, EP, WP and PN conceived and designed the experiments. JM, EP, WP, MS, TM, LY and CB performed the experiments. JM, EP, PN analyzed the data. JM and PN wrote the manuscript. All authors contributed to the article and approved the submitted version.
