# Supplementary material for: Assessing the adaptive role of cannabidiol (CBD) in Cannabis sativa defense against cannabis aphids
Source: Front Plant Sci. 2023 Oct 17;14:1223894. doi: 10.3389/fpls.2023.1223894 (PMC10616793; doi:10.3389/fpls.2023.1223894)
Supplement: Supplementary file 1 [file DataSheet_1.docx]

| Parameter  **Supplemental Table 1**. Modified from table in Güncan and Gümüş 2017 and Wei et al., 2020 | **Description** | **Formula** | **Explanation** | **References** |
| --- | --- | --- | --- | --- |
| l_x_ | Age-specific survival rate | $l_{x}=\sum_{j=1}^{k} s_{xj}$ | s_xj_ gives the survivorship probability for a newborn individual to age x and stage j, k = number of stages | Chi and Liu, 1985; Chi, 1988 |
| m_x_ | Age-specific fecundity | $m_{x}=\frac{\sum_{j=1}^{k} s_{xj}f_{xj}}{\sum_{j=1}^{k} s_{xj}}$ | f_xj_ is the age-stage-specific fecundity. This is the number of offspring produced by an individual at age x and stage j | Chi and Liu, 1985; Chi, 1988 |
| R_0_ | Net reproductive rate | $R_{0}=\sum_{x=0}^{\infty} l_{x}m_{x}$ | The number of offspring produced per female. | Chi and Liu, 1985; Chi, 1988 |
| r | Intrinsic rate of increase | $\sum_{x=0}^{\infty} e^{-r(x+1)}l_{x}m_{x}=1$ | Using Lotka formula to calculate a rate of increase per individual in an unlimited environment. | Lotka, 1913; Birch, 1948; Goodman, 1982; Chi and Liu, 1985; Chi, 1988 |
| λ | Finite rate of increase | $\lambda= e^{r}$ | The number of times a population multiplies in a unit of time. | Birch, 1948; Chi and Liu, 1985; Chi, 1988 |
| T | Mean generation time | $T= \frac{lnR_{0}}{r}$ | Time required for a population to reach R0 as λ is reached. | Chi and Liu, 1985; Chi, 1988 |
| e_xj_ | Age-stage-specific life expectancy | e_xj_ = $\sum_{i=x}^{\infty} \sum_{y=j}^{k} s'iy$ | Life expectancy for individual of age x and stage y. n is the number of age groups and m is the number of stages. S’ij is the probability that an individual of age x and stage y will survive to age I and stage j. Calculated assuming S’xy = 1. | Chi and Liu, 1985; Chi, 1988; Chi and Su, 2006 |
| v_xj_ | Age-stage reproductive value | $v_{xj}= \frac{e^{r(x+1)}}{s_{xj}} \sum_{i=x}^{\infty} e^{-r(i+1)} \sum_{y=i}^{m} {s'}_{iy}f_{iy}$ | The contribution of individuals at age x and stage j to the future population | Fisher, 1958; Huang and Chi, 2011; Tuan et al., 2014 |

| **Time (min)** | **Flow (mL/min)** | **%B** |
| --- | --- | --- |
| 0 | 0.2 | 59 |
| 5.3 | 0.2 | 59 |
| 7.05 | 0.2 | 73 |
| 9 | 0.2 | 73 |
| 12.2 | 0.2 | 99 |
| 12.3 | 0.3 | 99 |
| 13.6 | 0.3 | 99 |
| 13.7 | 0.2 | 59 |
| 16.5 | 0.2 | 59 |

**Supplemental Table 2.** LC-gradient for cannabinoids analysis in Table 2

**Supplemental Table 2.** LC-gradient for cannabinoids in Table 2

| **Target** | **Q1** | **Q3** | **Cone (V)** | **Collision energy (V)** | **Retention time (Min)** |
| --- | --- | --- | --- | --- | --- |
| CBDVA | 313.2 | 191.1 | 15 | 15 | 2.1 |
| CBDVA* | 313.2 | 233.1 | 15 | 15 | 2.1 |
| CBD | 315.2 | 123 | 25 | 35 | 4.69 |
| CBD* | 315.2 | 135.1 | 40 | 20 | 4.69 |
| CBG | 317.2 | 123 | 25 | 30 | 4.53 |
| CBG* | 317.2 | 193.1 | 25 | 15 | 4.53 |
| CBGA* | 343.2 | 219.1 | 15 | 15 | 4.25 |
| CBDA | 359.2 | 219.1 | 15 | 15 | 3.74 |
| CBDA* | 359.2 | 261.1 | 15 | 15 | 3.74 |
| CBGA | 361.2 | 261.1 | 15 | 15 | 4.25 |
| THCVA* | 313.2 | 191.1 | 45 | 25 | 5.83 |
| THCVA | 313.2 | 233.1 | 45 | 20 | 5.83 |
| delta9THC | 315.2 | 123 | 35 | 30 | 7.7 |
| delta9THC | 315.2 | 135.1 | 30 | 20 | 7.7 |
| delta9THC* | 315.2 | 193.1 | 35 | 20 | 7.7 |
| delta9THCA* | 341.2 | 219.1 | 45 | 25 | 8.56 |
| delta9THCA | 359.2 | 219.1 | 20 | 30 | 8.56 |
| CBCA/CBLA | 341.2 | 219.1 | 20 | 20 | 9.2 |
| CBCA/CBLA* | 359.2 | 219.1 | 20 | 25 | 9.2 |

Mobile phases were water with 0.1% formic acid (A) and acetonitrile (B). Samples were held at 6°C in the autosampler, and the column was operated at 45°C. Injection volume =2 µL.

**Supplemental Table 3.** MRM transitions for cannabinoids in Table 2

Note: CBCA and CBLA coelute, thus the data is the sum of two compounds. * denotes quantification transition

| **Time (min)** | **Flow (mL/min)** | **%B** |
| --- | --- | --- |
| 0 | 0.5 | 1 |
| 0.65 | 0.5 | 1 |
| 2.85 | 0.5 | 99 |
| 3.5 | 0.5 | 99 |
| 3.55 | 0.5 | 1 |
| 5 | 0.5 | 1 |

Mobile phases were water with 0.1% formic acid (A) and acetonitrile (B). Samples were held at 6°C in the autosampler, and the column was operated at 45°C. Injection volume =2 µL.

**Supplemental Table 4.** LC-gradient for phytohormone analysis in Figure 4

Supplementary Table Z MRM transitions for cannabinoids in Table 2

| **Target** | **Q1** | **Q3** | **Cone (V)** | **Collision energy (V)** | **Retention Time (Min)** |
| --- | --- | --- | --- | --- | --- |
| salicylic acid | 137.1 | 65 | 34 | 22 | 1.94 |
| salicylic acid | 137.1 | 93 | 34 | 16 | 1.94 |
| salicylic acid-D4 | 141.1 | 97 | 30 | 15 | 1.94 |
| abscisic acid | 263.2 | 153 | 32 | 10 | 1.96 |
| abscisic acid-D6 | 269.2 | 159 | 32 | 10 | 1.96 |
| jasmonic acid | 209.2 | 59 | 10 | 10 | 2.13 |
| jasmonic acid-D5 | 214.1 | 62 | 10 | 10 | 2.13 |

**Supplemental Table 5**. MRM transitions for phytohormone analysis in Figure 4

| Primer | Sequence | Size | Reference |
| --- | --- | --- | --- |
| CsClathrinF | TGTCAGTTTTGTGCCACCAG | 139 bp | Mangeot-Peter et al., 2016, |
| CsClathrinR | TCCATGCGTGTTCTACCAAG |  |  |
| HEL F | CATGGCGCAGCAAATATGG | 55 bp | Balthazar et al., 2020 |
| HEL R | CCCCTAGGTCCGGATGGT |  |  |
| PR1 F | GCGTAACTCGGTTCGTTTGG | 71 bp | Balthazar et al., 2020 |
| PR1 R | TGCAAGTGATGAAGGTACCCTTATT |  |  |
| CBDASqPCR_F | GCAATACACACTTACTTCTCTTCAGTTTTC | 241 bp | Fulvio et al., 2021 |
| CBDASqPCR_R | ACGTAGTCTAACTTATCTTGAAAGCAC |  |  |
| PP2C-6F | GACGACGACTGTCTGATTT | 134 bp | Gao et al., 2018 |
| PP2C-6R | GGTGATACCGAAGACGAG |  |  |

**Supplemental Table 6**. RT-qPCR primers used in this study

**References**

Supplementary Table 1 Modified from table in (Güncan and Gümüş 2017, Wei et al. 2020)

Balthazar, C., Cantin, G., Novinscak, A., Joly, D. L., Filion, M. (2020). Expression of putative defense responses in cannabis primed by *Pseudomonas* and/or *Bacillus* strains and infected by *Botrytis cinerea*. Front. Plant Sci. 11: 572112.

Birch, L.C. (1948). The intrinsic rate of natural increase in an insect population. J. Anim. Ecol. 17:1526.

Chi, H. (1988). Life-table analysis incorporating both sexes and variable development rates among individuals. Environ. Entomol. 17: 26–34.

Chi, H., and Liu, H. (1985). Two new methods for the study of insect population ecology. Bull. Inst. Zool. Acad. Sin. 24: 225–240.

Chi, H., and Su, H. Y. (2006). Age–stage, two-sex life tables of *Aphidius gifuensis* (Ashmead) (Hymenoptera: Braconidae) and its host *Myzus persicae* (Sulzer) (Homoptera: Aphididae) with mathematical proof of the relationship between female fecundity and the net reproductive rate. Environ. Entomol. 35: 10–21.

Fisher, R. A. (1958). The genetical theory of natural selection. New York: Dover Publication Inc.

Fulvio, F., Paris, R., Montanari, M., Citti, C., Cilento, V., Bassolino, L., Moschella, A., Alberti, I., Pecchioni, N., Cannazza, G., Mandolino, G. (2021). Analysis of sequence variability and transcriptional profile of cannabinoid synthase genes in *Cannabis sativa* L. chemotypes with a Focus on *Cannabichromenic acid synthase*. Plants. 10: 1857.

Gao, C., Cheng, C., Zhao, L., Yu, Y., Tang, Q., Xin, P., Liu, T., Yan, Z., Guo, Y., Zang, G. (2018). Genome-wide expression profiles of Hemp (*Cannabis sativa* L.) in response to drought stress. Int. J. Genomics. 3057272.

Goodman, D. (1982). Optimal life histories, optimal notation, and the value of reproductive value. Am. Nat. 119: 803–823.

Güncan, A., and Gümüş, E. (2017). Influence of different hazelnut cultivars on some demographic characteristics of the filbert aphid (Hemiptera: Aphididae*).*J. Econ. Entomol. 110: 1856–1862.

Huang, Y. B., and Chi, H. (2011). The age–stage, two-sex life table with an offspring sex ratio dependent on female age. J. Agric. For. 604: 337–345.

Lotka, A. J. (1913). A natural population norm. I. *J. Wash. Acad. Sci.* 3: 241–48.

Mangeot-Peter, L., Legay, S., Hausman, J. F., Esposito, S., Guerriero, G. (2016). Identification of reference genes for RT-qPCR data normalization in *Cannabis sativa* stem tissues. Int. J. Mol. Sci. 17: 1556.

Tuan, S. J., Lee, C., Chi, H. (2014). Population and damage projection of *Spodoptera litura* (F.) on peanuts (*Arachis hypogaea* L.) under different conditions using the age–stage, two-sex life table. Pest Manag. Sci. 70: 805–813.

Wei, M., Chi, H., Guo, Y., Li, X., Zhao, L., Ma, R. (2020). Demography of *Cacopsylla chinensis* (Hemiptera: Psyllidae) reared on four cultivars of *Pyrus bretschneideri* (Rosales: Rosaceae) and *P. communis* pears with estimations of confidence intervals of specific life table statistics. J. Econ. Entomol. 113: 2343 - 2353.
