# Supplementary figures and images for: Assessing the adaptive role of cannabidiol (CBD) in Cannabis sativa defense against cannabis aphids
Source: Front Plant Sci. 2023 Oct 17;14:1223894. doi: 10.3389/fpls.2023.1223894 (PMC10616793; doi:10.3389/fpls.2023.1223894)

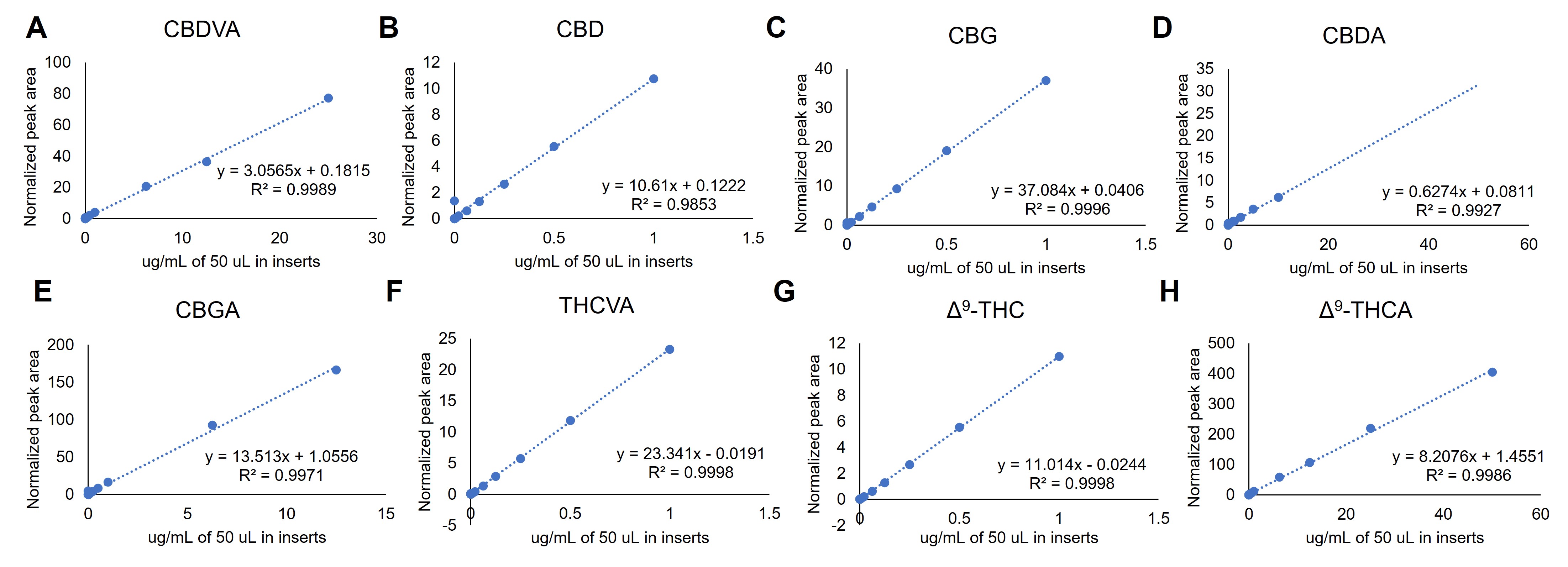

Supplement: Supplementary file 2 [file Image_1.jpeg]

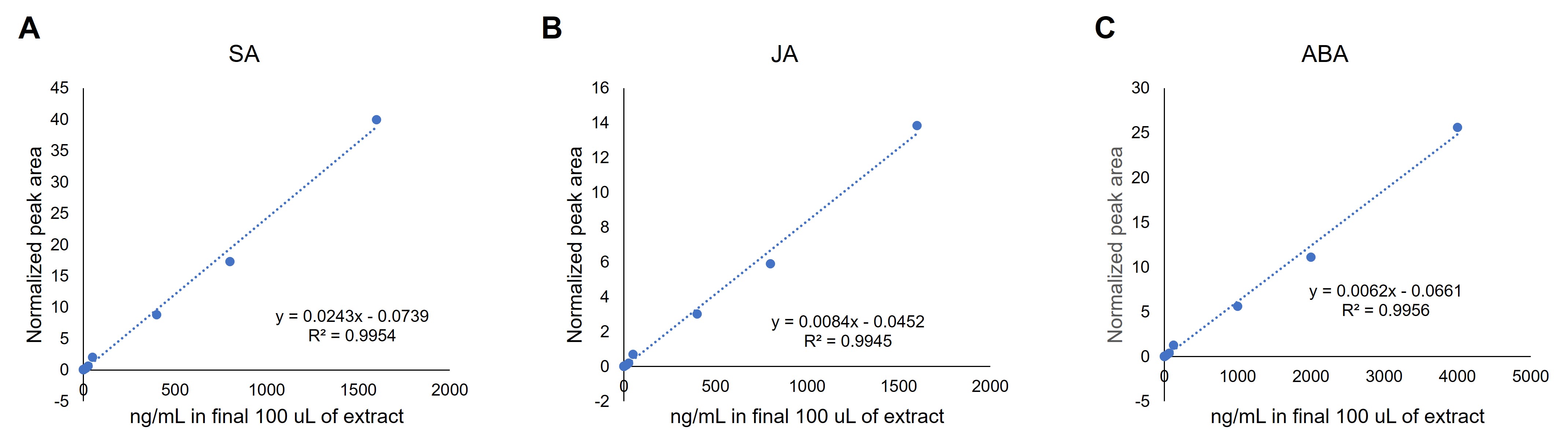

Supplement: Supplementary file 3 [file Image_2.jpeg]
